# Supplementary material for: Volatile Profile of Portuguese Monofloral Honeys: Significance in Botanical Origin Determination
Source: Molecules. 2021 Aug 17;26(16):4970. doi: 10.3390/molecules26164970 (PMC8400914; doi:10.3390/molecules26164970)
Supplement: Supplementary file 1 [file molecules-26-04970-s001.zip › molecules-1340142-supplementary.pdf]

molecules

Article

Volatile Profile of Portuguese Monofloral Honeys. Significance in Botanical Origin Determination

Alexandra M. Machado [1], Marília Antunes [2], Maria Graça Miguel [3], Miguel Vilas-Boas [4] and Ana Cristina Figueiredo [1,*]

1 Centro de Estudos do Ambiente e do Mar (CESAM Lisboa), Centro de Biotecnologia Vegetal (CBV), Faculdade de Ciências da Universidade de Lisboa, DBV, C2, Piso 1, Campo Grande, 1749-016 Lisboa, Portugal; ialexam@gmail.com
2 Centro de Estatística e Aplicações (CEAUL), Departamento de Estatística e Investigação Operacional, Faculdade de Ciências da Universidade de Lisboa, Campo Grande, 1749-016 Lisboa, Portugal; marilia.antunes@ciencias.ulisboa.pt
3 Faculdade de Ciências e Tecnologia, Mediterranean Institute for Agriculture, Environment and Development, Universidade do Algarve, Campus de Gambelas, 8005-139 Faro, Portugal; mgmiguel@ualg.pt
4 CIMO, Centro de Investigação de Montanha, Instituto Politécnico de Bragança, Campus de Santa Apolónia, 5300-253 Bragança, Portugal; mvboas@ipb.pt
* Correspondence: acsf@fc.ul.pt; Tel.: +351-217500257

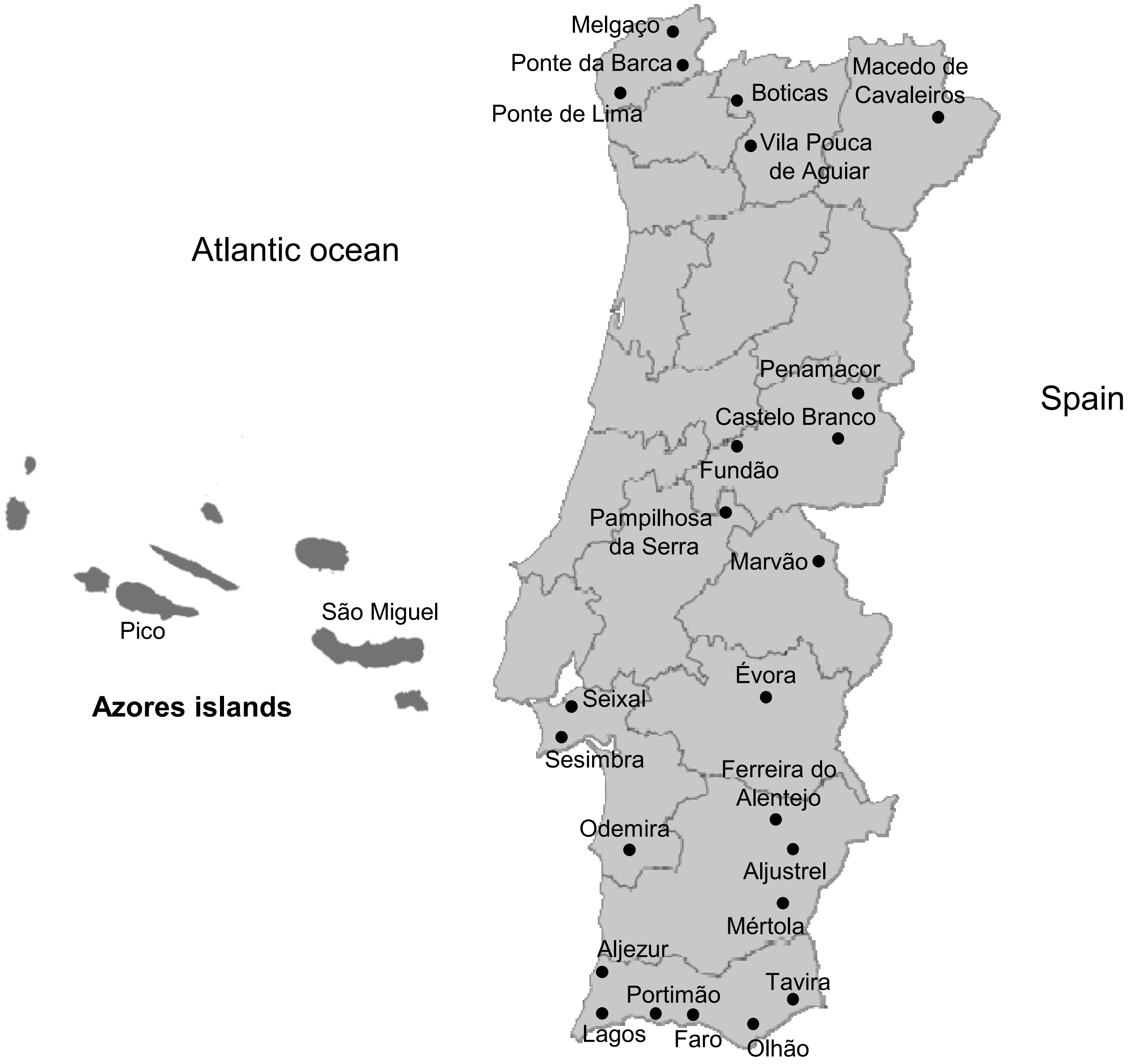

Figure S1. Geographical origin of the studied honey samples according to production locality.

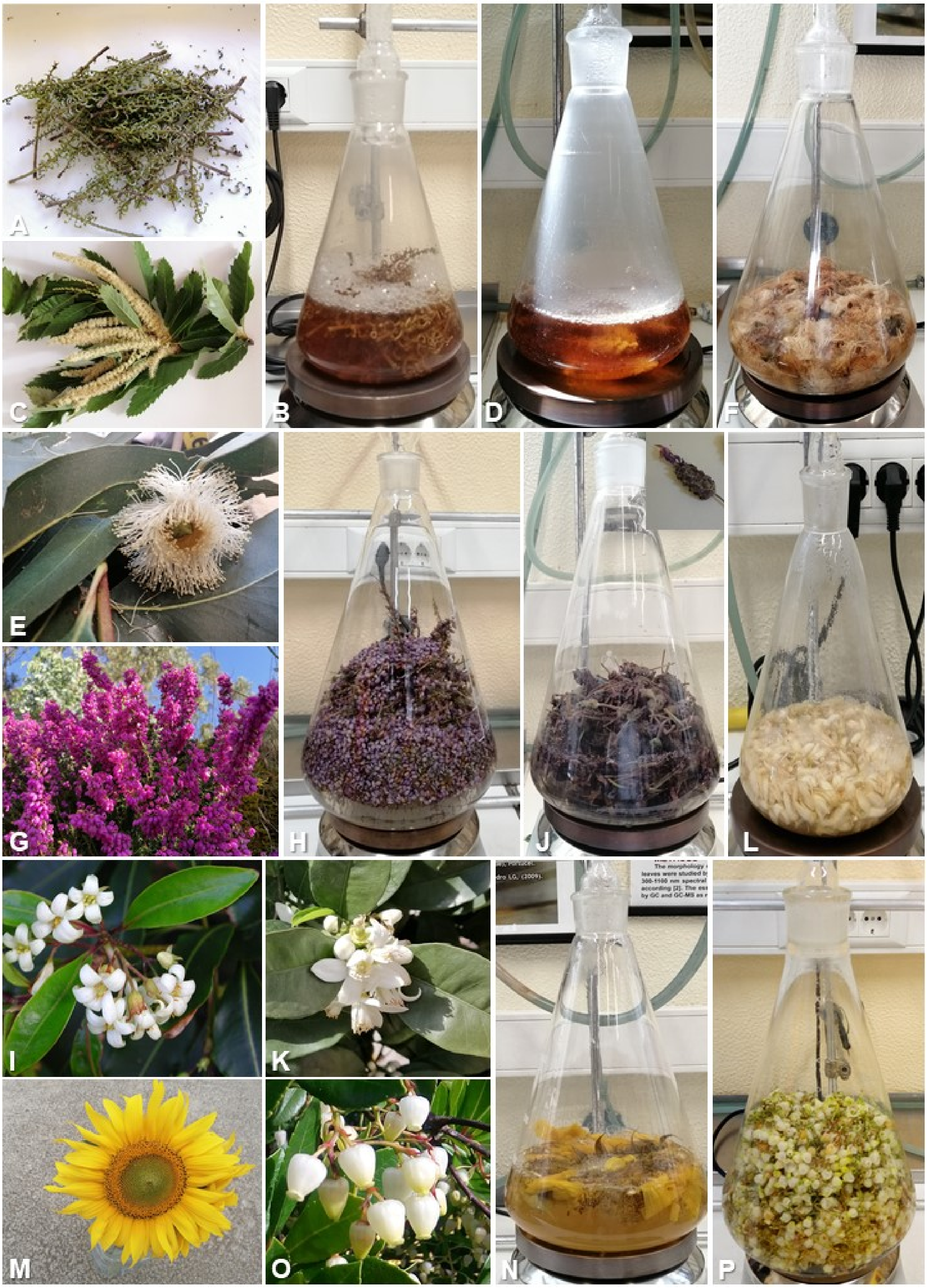

Figure S2. Detail of the flowers and extraction of flowers volatiles by hydrodistillation. A-B. Carob tree. C-D. Chestnut. E-F. Eucalyptus. G-H. Bell heather. I. Incense. J. Lavender. K-L. Orange. M-N. Sunflower. O-P. Strawberry tree.

molecules

MDPI

Table S1. Volatile profile of the honey samples obtained by solid phase microextraction (SPME). The minimum and maximum values represent the lowest and highest percentages, respectively, of each identified component.

Components	RI	Honeys																						
		Carob tree		Chestnut		Eucalyptus		Bell Heather		Incense		Lavender		Orange		Rape		Raspberry		Rosemary	Sunflower		Strawberry tree	
		Ct1-Ct5		C1-C2		E1-E5		H1-H6		I1-I4		L1-L8		O1-O9		R1-R2		Rb1-Rb2		Ro1	Sf1-Sf3		St1-St4	
		Min	Max	Min	Max	Min	Max	Min	Max	Min	Max	Min	Max	Min	Max	Min	Max	Min	Max	Min/Max	Min	Max	Min	Max
Acetaldehyde	497		t	t	t	t	t		t	t	27.6	t	0.8			4.9	5.5							
n-Hexane	600																				t	6.8		
2-Furfural	825	t	t						t	t	6.8		t		t								t	t
Isovaleric acid	867														t				7.1					
5-Methyl-3-hexen-2-one *	889																						t	t
2-Acetyl furan *	897																						t	t
2,6-Dimethylpyrazine *	899																						t	0.1
Benzaldehyde	927		t							t	32.5			t	7.1		t		2.8				t	0.1
5-Methyl furfural	938														t									
3-Methyl valeric acid *	947																		5.7					
Hexyl acetate	995														1.2	t	t		t		t	t	t	t
Benzyl alcohol	1000									1.6	10.7													
Benzene acetaldehyde	1002		t						t	t	29.2		t		6.2				1.9					
1,8-Cineole	1005																				t	t		
cis-Linalool oxide (furanoid)	1045		32.2				14.7	t	26.7	7.6	47.3			t	8.8	t	t		4.2					
trans-Linalool oxide (furanoid)	1059		8.3				t	t	t	t	12.5			t	3.7									
Heptanoic acid	1064	0.3	3.7		t				t	8.3	30.6		t		2.3				8.7					
Phenyl ethyl alcohol	1067									t	8.6													
n-Nonanal	1073	0.2	3.7	t	t		t		t				0.4			2.2	3.0				1.8	3.7		
Hotrienol *	1074		0.2				t		t						t		t		t	0.1				
α-Isophorone * (2-cyclohexen-1-one, 3,5,5-trimethyl-)	1074		8.6												6.1	t	2.6				t	t	49.8	72.6

Components	RI	Honeys																						
		Carob tree		Chestnut		Eucalyptus		Bell Heather		Incense		Lavender		Orange		Rape		Raspberry		Rosemary	Sunflower		Strawberry tree	
		Ct1-Ct5		C1-C2		E1-E5		H1-H6		I1-I4		L1-L8		O1-O9		R1-R2		Rb1-Rb2		Ro1	Sf1-Sf3		St1-St4	
		Min	Max	Min	Max	Min	Max	Min	Max	Min	Max	Min	Max	Min	Max	Min	Max	Min	Max	Min/Max	Min	Max	Min	Max
4-Keto-isophorone * (2-cyclohexene-1,4-dione, 2,6,6-trimethyl-)	1101		0.9					t	2.8						t		t				t	t	3.2	4.1
Lilac aldehyde A *	1102		2.7												4.5				8.0	t				
Lilac aldehyde B *	1109		1.3												9.0				12.8					
2-Hydroxyisophorone * (2-hydroxy-3,5,5-trimethyl-2-cyclohex-1-enone)	1114																						1.3	4.0
Lilac aldehyde C *	1123		2.1												4.4				7.0					
UI 1	1128																			1.0			t	1.0
UI 2	1128																						t	1.0
cis-Linalool oxide (pyranoid) *	1132		2.9																					
Lilac aldehyde D *	1138														3.1									
trans-Linalool oxide (pyranoid) *	1143		4.5																					
UI 3	1157	2.3	5.4				t						t											
5-Hydroxymethyl furfural	1158									t	t													
Lilac aldehyde E *	1165														1.2									
Butyl hexanoate *	1173															t	t			t				
Ethyl octanoate	1177														t					0.2				
n-Decanal	1180		16.6	t	t	t	t	t	7.7			t	t	t	3.6	2.6	2.7				2.0	3.3	0.3	1.1
Diacetin	1227								t				t											
2-*trans*-Decenal	1236															2.5	2.8							
2,3,5-Trimethylphenol (isopseudocumenol)	1252																			t			t	0.8
trans-Anethole	1254															t	t							
1-Decanol	1259														3.0					t				
Thymol	1275															t	t							

Components	RI	Honeys																						
		Carob tree		Chestnut		Eucalyptus		Bell Heather		Incense		Lavender		Orange		Rape		Raspberry		Rosemary	Sunflower		Strawberry tree	
		Ct1-Ct5		C1-C2		E1-E5		H1-H6		I1-I4		L1-L8		O1-O9		R1-R2		Rb1-Rb2		Ro1	Sf1-Sf3		St1-St4	
		Min	Max	Min	Max	Min	Max	Min	Max	Min	Max	Min	Max	Min	Max	Min	Max	Min	Max	Min/Max	Min	Max	Min	Max
3,4,5-Trimethylphenol (3,4,5-hemimellitenol)	1277		2.5																					
UI 4	1280																			0.9			0.5	0.8
n-Undecanal	1288		1.7												2.8					0.3			1.7	6.1
UI 5	1293	1.2	2.4										t											
Methyl anthranilate	1300														4.3									
Triacetin	1316		81.8	t	t	t	t	t	65.3		t		79.1		70.9	12.0	17.9		5.7		8.7	27.0	0.3	13.6
Edulan *	1345																						4.4	6.5
Hexyl hexanoate *	1375														0.6						t	1.1	t	t
UI 6	1384							t	7.1															
Ethyl decanoate	1387														1.2									
n-Tetradecane	1400	t	2.5	t	3.8	t	15.1	1.7	4.7			t	4.5		6.1	2.7	4.8		3.0		3.9	5.0	0.3	1.5
Geranyl acetone	1434		13.1	t	t		8.0		t			t	t		2.6	t	0.9		t		t	3.5	0.1	1.0
α-*trans*, *trans*-Farnesene	1500		t												4.8	t	t				t	6.4	0.2	2.3
n-Pentadecane	1500	0.6	3.6			t	9.8	t	4.2				4.2	t	6.9	7.3	10.4		3.2		t	3.4	t	t
2,5-Furandicarboxaldehyde	1511		t							t	t		t	t	3.8				5.4				t	0.2
Benzophenone *	1577														1.8								t	0.2
n-Hexadecane	1600	0.6	1.9	t	t	t	14.2	1.3	5.1			t	5.1		8.6	2.4	3.3		2.2		2.8	3.2	0.2	1.3
cis-Methyl dihydrojasmonate	1616		0.9	t	t		0.5		21.9				13.9								t	t		
UI 7	1652		1.2				t		t						48.2	37.1	37.4				37.8	54.3	0.4	9.1
UI 8	1667		10.3			16.5	34.8	5.8	41.3			2.7	43.2						17.1					
n-Heptadecane	1700	0.6	3.0	t	t	t	5.2	t	1.1				4.5	t	32.4	9.5	11.0	2.5	14.3		t	4.7	t	1.5
Octyl salicylate *	1743		6.8	40.6	58.8	28.7	40.4	t	35.7			4.0	50.9						25.4					
n-Octadecane	1800			t	t		t		t				0.7			t	t			0.1				t
Isopropyl tetradecanoate *	1803					t	3.1		2.4				t			1.0	1.6							
1-Hexadecanol	1821								t												t	t		
n-Nonadecane	1900		2.6										t		17.6	2.2	2.9		4.6		t	t		0.1
**% Identification**		58.2	90.7	44.4	58.8	49.6	69.7	50.6	83.2	72.2	100.0	13.1	89.8	47.4	90.9	58.3	60.4	42.0	82.5	61.9	40.7	48.4	82.7	93.8

Components	RI	Honeys																						
		Carob tree		Chestnut		Eucalyptus		Bell Heather		Incense		Lavender		Orange		Rape		Raspberry		Rosemary	Sunflower		Strawberry tree	
		Ct1-Ct5		C1-C2		E1-E5		H1-H6		I1-I4		L1-L8		O1-O9		R1-R2		Rb1-Rb2		Ro1	Sf1-Sf3		St1-St4	
		Min	Max	Min	Max	Min	Max	Min	Max	Min	Max	Min	Max	Min	Max	Min	Max	Min	Max	Min/Max	Min	Max	Min	Max
**Grouped components**																								
**Terpenes and derivatives**																								
Oxygen-containing monoterpenes		3.4	51.4				14.7	t	26.7	7.6	47.3			t	32.7	2.5	2.8		32.0	t	t	t		
Sesquiterpene hydrocarbons			t												4.8	t	t			t	t	6.4	0.2	2.3
Apocarotenoids			13.1	t	t		8.0	t	2.8			t	t	t	6.1	0.9	2.6		t		t	3.5	59.7	87.5
**Amino acid derivatives and Phenylpropanoids**																								
Benzoic acids derivatives		t	6.8	40.6	58.8	28.7	40.4	t	35.7	1.6	43.2	4.0	50.9	t	7.1		t	2.8	25.4				t	0.1
Phenylpropenes		0.3	3.7		t				t	8.3	30.6		t		2.3				8.7					
Aromatic amino acid derivatives			1.7						t	t	29.2		t	t	6.2				1.9				1.7	6.9
**Fatty acids and derivatives**																								
Green leaf volatiles (GLV)															1.2	t	t		t	t	t	t	t	t
Fatty acids										t	8.6				t				12.8					
Alkanes		3.3	11.0	t	3.8	3.2	34.4	4.7	14.0			1.2	13.1	4.1	50.0	27.8	28.7	10.9	18.9	2.1	9.1	17.3	0.7	4.3
Other fatty acid derivatives		7.6	82.2	t	t	t	3.1	t	65.3		t	t	79.1	t	72.2	18.7	24.3		5.7	8.2	15.7	31.1	0.9	14.7
**Carbohydrate derivatives**		t	t						t	t	6.8		t	t	3.8				5.4				t	0.2
**Nitrogen containing compounds**																							t	0.1
Others			t	t	t	t	t		t	t	27.6	t	0.8		1.8	4.9	5.5			51.6			t	0.2

RI: In-lab calculated retention index relative to C6-C31 *n*-alkanes on the DB-1 column. Min: Minimum. Max: Maximum. t: traces (< 0.05 %). * Identification based on mass spectra only. UI: unidentified compounds. Ct: Carob tree. C: Chestnut. E: Eucalyptus. H: Bell heather. I: Incense. O: Orange. R: Rape. Rb: Raspberry. Ro: Rosemary. L: Lavender. S: Sunflower. St: Strawberry tree.

Table S2. Volatile profile of the honey samples obtained by hydrodistillation (HD). The minimum and maximum values represent the lowest and highest percentages, respectively, of each identified component.

Components	RI	Honeys																						
		Carob tree		Chest-nut		Eucalyp-tus		Bell Heather		In-cense		Laven-der		Orange		Rape		Raspber-ry		Rose-mary	Sunflow-er		Strawber-ry tree	
		Ct1-Ct5		C1-C2		E1-E5		H1-H6		I1-I4		L1-L8		O1-O9		R1-R2		Rb1-Rb2		Ro1	Sf1-Sf3		St1-St4	
		Min	Max	Min	Max	Min	Max	Min	Max	Min	Max	Min	Max	Min	Max	Min	Max	Min	Max	Min/Max	Min	Max	Min	Max
n-Heptane	700												0.2											
n-Hexanal	739		t		t		0.1						t											
n-Octane	800		0.3		t		0.5		0.2		0.1		0.4		0.3			0.1	0.3	0.1				0.5
2-Furfural	825		2.0		t	0.6	1.6	0.1	2.3	0.1	0.6		1.1	t	0.7	t	0.1	t	0.6	0.1	0.1	0.2	t	0.6
Ethylbenzene	843															t	t				t	t		
Furfuryl alcohol	855						0.1		0.2															
Isovaleric acid	867		0.2			t	0.5		0.3				t					0.1	0.2					
2-Methylbutyric acid	871		0.3		t	t	0.6		0.2									t	t					
n-Hexanol	881						t		0.1										t					
Valeric acid (pentanoic acid)	888						t				t													
5-Methyl-3-hexen-2-one *	889						t																t	0.4
n-Heptanal	897					t	t						t											
2-Acetyl furan *	897		0.1		t	t	0.1		0.2		t		t						t			t	t	0.1
2,6-Dimethylpyrazine *	899																						t	0.1
n-Nonane	900		t			t	0.1		0.2		0.1		0.1		t				t			t		t
2-Methyl-1-octen-3-yne	917								1.2															
UI 1	918																							t
Benzaldehyde	927		0.2	0.4	1.2		0.9	0.1	1.7	t	0.4		1.1		0.2	t	t	t	0.2	0.1		t		0.1
α-Pinene	930		0.4				0.1		0.6				0.3		0.2			t	0.2		t	0.2		0.1
5-Methyl furfural	938		0.2				0.4		0.3		0.1		t		t								t	0.1
Dimethyl trisulfide	956															t	t							
β-Pinene	963		t				0.1		0.1				0.1		t									
Phenol	968						t	t	0.2															
Hexanoic acid	968		0.6		0.1		0.1		0.2	t	0.1	t	0.1		t			t	0.1	t		t	t	0.1
n-Octanal	973		t				t		t				0.1						0.1			t		

Components	RI	Honeys																						
		**Carob tree**		**Chestnut**		**Eucalyptus**		**Bell Heather**		**Incense**		**Lavender**		**Orange**		**Rape**		**Raspberry**		**Rosemary**	**Sunflower**		**Strawberry tree**	
		**Ct1-Ct5**		**C1-C2**		**E1-E5**		**H1-H6**		**I1-I4**		**L1-L8**		**O1-O9**		**R1-R2**		**Rb1-Rb2**		**Ro1**	**Sf1-Sf3**		**St1-St4**	
		**Min**	**Max**	**Min**	**Max**	**Min**	**Max**	**Min**	**Max**	**Min**	**Max**	**Min**	**Max**	**Min**	**Max**	**Min**	**Max**	**Min**	**Max**	**Min/Max**	**Min**	**Max**	**Min**	**Max**
β-Myrcene	975		t				t		0.1		t		0.2		0.1							0.1		t
n-Decane	1000					t	0.1		t				t									t		t
Benzyl alcohol	1000						0.4	t	0.9		t													0.1
Benzene acetaldehyde	1002	t	1.1	2.1	2.2		2.6	1.9	24.4	1.0	3.1	3.5	9.9	t	1.4	0.1	0.4	1.2	3.7	1.0	0.1	0.7	t	0.3
p-Cymene	1003						0.2				0.1				0.3							t		
2,6,6-Trimethylcyclohexanone	1003								t						t							t		
2-Ethyl 1-hexanol *	1004		t		t	t	t	t	t	t	t	t	t	t	t	t	t	t	t	t	t	t		
1,8-Cineole	1005		t		t				0.1		0.1		0.2	t	0.3	t	t		0.3	0.2		t		0.2
Limonene	1009		t				t		0.3		0.3		t						t					
Acetophenone	1017		t	0.1	0.4		t	t	0.5				0.4		t				t			t		
6-Heptenoic-acid	1019								0.1															
γ-Terpinene	1035						t								0.2									
cis-Linalool oxide (furanoid)	1045	0.1	15.1	t	0.6	0.2	3.9	2.2	11.9	1.0	2.9		0.5	0.1	0.7	t	0.1	0.2	0.3	0.1		t		0.1
trans-Linalool oxide (furanoid)	1059	t	5.1	t	0.3	0.2	2.1	1.0	4.6	0.6	1.7		t	t	0.3		t	t	0.2	t				t

Components	RI	Honeys																						
		Carob tree		Chest-nut		Eucalyp-tus		Bell Heather		In-cense		Laven-der		Orange		Rape		Raspber-ry		Rose-mary	Sunflow-er		Strawber-ry tree	
		Ct1-Ct5		C1-C2		E1-E5		H1-H6		I1-I4		L1-L8		O1-O9		R1-R2		Rb1-Rb2		Ro1	Sf1-Sf3		St1-St4	
		Min	Max	Min	Max	Min	Max	Min	Max	Min	Max	Min	Max	Min	Max	Min	Max	Min	Max	Min/Max	Min	Max	Min	Max
Heptanoic acid	1064						0.101																	
Phenyl ethyl alcohol	1064		0.9		0.1		0.2		1.1	t	0.3		1.0	t	0.3					t				t
n-Nonanal	1073		t		0.1	0.1	0.4		0.2		0.4		0.8				t	0.2	0.6		t	0.1		t
Linalool	1074		0.2		0.1		0.4			t	0.5				0.3									
Hotrienol *	1074		2.9	0.1	0.8	t	5.1	2.3	9.8				0.7			0.1	0.4	0.3	0.3	0.3		0.1		
α-Isophorone * (2-cyclohexen-1-one, 3,5,5-trimethyl-)	1074		0.1				0.2		2.6						0.1		t					0.1	4.5	38.8
UI 2	1100		t						t											0.9				
n-Undecane	1100		t		t	t	0.1		t		t		0.1		t				t			t		
4-Keto-isophorone * (2-cyclohexene-1,4-dione, 2,6,6-trimethyl-)	1101		t		0.1	t	0.4	0.1	0.6						t							t	0.4	2.7
Cosmene *	1102		t				0.1	t	0.7				t											
Lilac aldehyde A *	1102		0.4				0.4						0.2		0.8			0.1	0.2					
Lilac aldehyde B *	1109		0.4				0.2		0.3				t		1.3			0.1	0.1					
cis-Verbenol	1113						t		0.1															
2-Hydroxyisophorone *	111						1.4															t	0.2	3.0

Components	RI	Honeys																						
		Carob tree		Chestnut		Eucalyptus		Bell Heather		Incense		Lavender		Orange		Rape		Raspberry		Rosemary	Sunflower		Strawberry tree	
		Ct1-Ct5		C1-C2		E1-E5		H1-H6		I1-I4		L1-L8		O1-O9		R1-R2		Rb1-Rb2		Ro1	Sf1-Sf3		St1-St4	
		Min	Max	Min	Max	Min	Max	Min	Max	Min	Max	Min	Max	Min	Max	Min	Max	Min	Max	Min/Max	Min	Max	Min	Max
(2-hydroxy-3,5,5-trimethyl-2-cyclohex-1-enone)	4																							
Lilac aldehyde C *	1123		0.1										t		0.7			t	t					
2-*trans*-Nonen-1-al	1124								0.1															
Nerol oxide	1127		0.3				0.4	t	0.5				t											
UI 3	1128						0.3		0.1														0.1	1.0
UI 4	1128																						0.1	1.0
cis-Linalool oxide (pyranoid) *	1132		0.3				0.1		0.2															
Benzoic acid	1132								t															
Lilac aldehyde D *	1138														0.2									
Veratrole * (1,2-dimethoxybenzene)	1139																							2.1
trans-Linalool oxide (pyranoid) *	1143		0.6				0.1	t	0.1															
1-Nonanol	1148			0.1	0.1	t	0.6		t				0.1											
p-Cymen-8-ol	1148						0.2																	
Octanoic acid	114		0.5	t	0.3	0.1	1.0	0.1	0.9		t		0.1		0.1		t	t	0.2	0.1	t	t	t	0.2

Components	RI	Honeys: Carob tree Ct1-Ct5 Min	Max	Chestnut C1-C2 Min	Max	Eucalyptus E1-E5 Min	Max	Bell Heather H1-H6 Min	Max	Incense I1-I4 Min	Max	Lavender L1-L8 Min	Max	Orange O1-O9 Min	Max	Rape R1-R2 Min	Max	Raspberry Rb1-Rb2 Min	Max	Rosemary Ro1 Min/Max	Sunflower Sf1-Sf3 Min	Max	Strawberry tree St1-St4 Min	Max
	9																							
UI 5	1157		0.2																					
UI 6	1157						0.5	t	1.4															
α-Terpineol	1159		t				0.1		t	t	0.3		t											
Safranal	1160																							0.1
Lilac aldehyde E *	1165														t									
UI 7	1165		0.8				0.1																	
Myrtenol	1168						t		0.2															
n-Decanal	1180		t		0.1		0.1		2.4				0.4						0.3		t	t		
UI 8	1180		0.4			t	0.1		0.2		t		t	t	1.4		t	0.1	0.2					
UI 9	1181		0.4			t	0.2		0.2		0.1		t	t	1.3		t	0.1	0.2					
2′-Hydroxyacetophenone	1188								0.7															
n-Dodecane	1200						0.1		t				t						t			t		
α-Fenchyl acetate	1200																					0.2		

Components	RI	Honeys																						
		Carob tree		Chest-nut		Eucalyp-tus		Bell Heather		In-cense		Laven-der		Orange		Rape		Raspber-ry		Rose-mary	Sunflow-er		Strawber-ry tree	
		Ct1-Ct5		C1-C2		E1-E5		H1-H6		I1-I4		L1-L8		O1-O9		R1-R2		Rb1-Rb2		Ro1	Sf1-Sf3		St1-St4	
		Min	Max	Min	Max	Min	Max	Min	Max	Min	Max	Min	Max	Min	Max	Min	Max	Min	Max	Min/Max	Min	Max	Min	Max
cis-Myrcenol	1205														0.2									
p-Anisaldehyde	1210						0.1		0.8				t									t		
Phenyl acid ethyl ester	1210								t															
trans-Cinnamaldehyde	1224			0.1	0.4		0.4		t		t		0.3						0.2					
Phellandral *	1240						0.1																	
Indole	1251														0.3									
2,3,5-Trimethylphenol (isopseudocumenol)	1252		0.2				0.1		0.2				0.4		0.3				0.2			0.4	0.4	3.8
2'-Aminoacetophenone	1253			t	0.7				t															
UI 10	1254		0.1				0.1		1.3				1.6									t		t
Nonanoic acid	1263		0.4		0.2	0.1	1.4	t	1.3	t	0.1		0.3	t	0.4			t	t	t	t	t	t	t
UI 11	1265		t				0.2		0.3															
Bornyl acetate	1265																					0.1		
Cumin alcohol	1265						0.3																	
Dihydrosafrole	127		0.2				t						t											

Components	RI	Honeys																						
		Carob tree		Chest-nut		Eucalyp-tus		Bell Heather		In-cense		Laven-der		Orange		Rape		Raspber-ry		Rose-mary	Sunflow-er		Strawber-ry tree	
		Ct1-Ct5		C1-C2		E1-E5		H1-H6		I1-I4		L1-L8		O1-O9		R1-R2		Rb1-Rb2		Ro1	Sf1-Sf3		St1-St4	
		Min	Max	Min	Max	Min	Max	Min	Max	Min	Max	Min	Max	Min	Max	Min	Max	Min	Max	Min/Max	Min	Max	Min	Max
	1																							
Perillyl alcohol	1274								0.2															
2-Undecanone	1275						t						0.1											
Thymol	1275		t				t		0.2				0.2											
3,4,5-Trimethylphenol (3,4,5-hemimellitenol)	1277		0.3	t	0.1	t	0.5		0.6				0.7		0.2		t					t	1.5	13.3
UI 12	1280						0.5		0.1														t	0.3
4-Vinylguaiacol (2-methoxy-4-vinylphenol)	1285				t	t	t		t						t									t
Carvacrol	1286		0.1				t		0.1		t				0.2									0.4
2-Undecanol *	1288						0.3																	
UI 13	1293		t																					
Methyl anthranilate	1300		0.3										t		2.1			t	0.1					
n-Tridecane	1300		0.2		0.2	0.2	0.4		0.1	t	0.1	t	0.3	t	t	t	t	0.1	0.1	t	t	0.2		
1,2-Dihydro-1,1,6-trimethyl-naphtalene *	1328		t			t	0.2		3.1				0.1						t				t	1.0
Edulan *	1345						0.2																t	0.7

Components	RI	Honeys																						
		Carob tree		Chest-nut		Eucalyp-tus		Bell Heather		In-cense		Laven-der		Orange		Rape		Raspber-ry		Rose-mary	Sunflow-er		Strawber-ry tree	
		Ct1-Ct5		C1-C2		E1-E5		H1-H6		I1-I4		L1-L8		O1-O9		R1-R2		Rb1-Rb2		Ro1	Sf1-Sf3		St1-St4	
		Min	Max	Min	Max	Min	Max	Min	Max	Min	Max	Min	Max	Min	Max	Min	Max	Min	Max	Min/Max	Min	Max	Min	Max
trans-3-Decenoic acid	1345								0.2															
Decanoic acid	1356	t	0.3	0.2	0.7		2.2	0.3	6.4		t		0.3		0.1			t	0.1	t	t	t	t	0.4
trans-β-Damascenone	1372		0.5				0.1		0.1															
α-Isocomene	1375												0.2											
UI 14	1384						0.3	0.1	1.5															
Dodecanal	1397						0.1																	
α-Gurjunene	1400						0.1																	
n-Tetradecane	1400		t		t	t	0.2	t	0.1		t	t	0.1	t	t	t	t	t	0.1		t	t		
p-Anisic acid ethyl ester * (ethyl anisate)	1414								0.1															
β-Caryophyllene	1414						t						1.2									0.1		
β-Copaene *	1426																				0.1	0.2		
Aromadendrene	1428						0.4		0.2										t					
γ-Decalactone	1430						0.2		0.7															
Geranyl acetone	143		t				t						t								t	t		

Components	RI	Honeys																						
		Carob tree		Chest-nut		Eucalyp-tus		Bell Heather		In-cense		Laven-der		Orange		Rape		Raspber-ry		Rose-mary	Sunflow-er		Strawber-ry tree	
		Ct1-Ct5		C1-C2		E1-E5		H1-H6		I1-I4		L1-L8		O1-O9		R1-R2		Rb1-Rb2		Ro1	Sf1-Sf3		St1-St4	
		Min	Max	Min	Max	Min	Max	Min	Max	Min	Max	Min	Max	Min	Max	Min	Max	Min	Max	Min/Max	Min	Max	Min	Max
	4																							
δ-Decalactone	1452						t	t	0.6															
allo-Aromadendrene	1456						0.2																	
UI 15	1457	t	0.1		t		0.1		0.2		t	t	0.2	t	0.2		t	t	0.1	0.1		0.1		
Dodecanol	1468						t		0.3														0.1	0.2
Germacrene D	1474																				t	0.1		
Zingiberene	1492								0.1															
UI 16	1493						t								t									
Tridecanal	1499				t														0.1					
α-trans, trans-Farnesene	1500		t				0.1		0.2		t		0.5		t							t		
β-Bisabolene	1500						0.1				0.1		0.7		t		t					0.1		
n-Pentadecane	1500	0.5	1.2	0.7	1.3	0.9	1.9	0.2	0.7	0.4	1.6	0.5	1.7	0.4	1.4	1.0	1.6	0.9	1.0	1.0	0.7	0.8	0.2	0.9
trans-Calamenene	1505								t		t													
δ-Cadinene	1505		t				t		t		0.2		t								t	t		t

Components	RI	Honeys																						
		Carob tree		Chest-nut		Eucalyp-tus		Bell Heather		In-cense		Laven-der		Orange		Rape		Raspber-ry		Rose-mary	Sunflow-er		Strawber-ry tree	
		Ct1-Ct5		C1-C2		E1-E5		H1-H6		I1-I4		L1-L8		O1-O9		R1-R2		Rb1-Rb2		Ro1	Sf1-Sf3		St1-St4	
		Min	Max	Min	Max	Min	Max	Min	Max	Min	Max	Min	Max	Min	Max	Min	Max	Min	Max	Min/Max	Min	Max	Min	Max
2,5-Furandicarboxaldehyde	1511									t	t													
UI 17	1511				0.1		0.1	t	0.8															
α-Calacorene	1525		t		t		t		0.1		t		t		t							t		
Dodecanoic acid	1550		0.1	0.1	0.1		0.3	t	0.4	t	t		0.7		0.2				t	t	0.2	0.5	t	0.4
β-Caryophyllene oxide	1561												t											
Globulol	1566				0.6		t												0.1					
Ethyl dodecanoate (ethyl laurate)	1580												0.2											
5-*epi*-7-*epi*-α-Eudesmol	1596				t										0.2									0.6
Tetradecanal	1596		t		t		0.1		t															
n-Hexadecane	1600	0.1	0.2	0.1	0.2	t	0.4	t	1.1	t	0.1	t	0.4	t	0.2	0.2	0.3	0.1	0.1	0.2	0.1	0.1	t	0.1
UI 18	1609														0.3									1.5
ɣ-Eudesmol	1609			t	0.4	0.1	0.5		0.2	t	0.1		0.4	t	0.7	t	t	0.4	0.6	t	t	t		0.1
T-Cadinol (*epi*-α-cadinol)	1616						0.3						0.2		0.3									2.9
δ-Cadinol (α-muurolol)	161		t				t		t															

Components	RI	Carob tree Ct1-Ct5 Min	Max	Chestnut C1-C2 Min	Max	Eucalyptus E1-E5 Min	Max	Bell Heather H1-H6 Min	Max	Incense I1-I4 Min	Max	Lavender L1-L8 Min	Max	Orange O1-O9 Min	Max	Rape R1-R2 Min	Max	Raspberry Rb1-Rb2 Min	Max	Rosemary Ro1 Min/Max	Sunflower Sf1-Sf3 Min	Max	Strawberry tree St1-St4 Min	Max
	8																							
β-Eudesmol	1620				0.2	0.1	0.4		0.1		t		0.1		0.1			0.2	0.3		t	t		0.1
α-Cadinol	1626				0.3				t						0.1									0.5
α-Eudesmol	1634					0.1	0.5		0.2		0.4				0.2			0.2	0.3		t	t		
UI 19	1648																							0.5
UI 20	1652		t		0.1		0.3		0.4		0.1		0.3		t		t				t	0.4		
UI 21	1656	0.6	1.6	0.2	1.3	0.5	1.6	0.3	0.9	0.1	1.1	0.2	1.3	0.4	1.3	0.4	0.6	0.2	0.7	0.6	0.5	0.6	0.1	1.1
UI 22	1661	0.2	1.2	0.1	0.3	0.1	0.4	0.1	0.6		0.1	t	1.0	0.1	0.8	0.5	0.6	0.4	0.8	0.5	0.3	0.8		0.3
Heptadecene *	1668	0.9	2.7	0.9	2.0	1.1	2.0	t	1.4	0.3	1.5	0.1	2.4	0.8	2.4	1.1	1.3	1.0	2.0	1.1	0.6	1.1	0.2	1.5
Pentadecanal	1688				0.1																			
n-Heptadecane	1700	1.7	5.4	2.0	4.2	2.1	5.9	1.0	2.9	0.8	2.6	1.0	5.9	1.2	5.5	2.9	7.9	2.3	5.0	3.4	2.0	2.9	0.3	3.0
Ambroxide *	1714		t				t								0.1				t					
Tetradecanoic acid (myristic acid)	1723		t				t		t	t	0.2		t								1.5	2.0	t	0.3
Octyl salicylate *	1743		0.1						t		0.4				0.1									

Components	RI	Honeys																						
		Carob tree		Chestnut		Eucalyptus		Bell Heather		Incense		Lavender		Orange		Rape		Raspberry		Rosemary	Sunflower		Strawberry tree	
		Ct1-Ct5		C1-C2		E1-E5		H1-H6		I1-I4		L1-L8		O1-O9		R1-R2		Rb1-Rb2		Ro1	Sf1-Sf3		St1-St4	
		Min	Max	Min	Max	Min	Max	Min	Max	Min	Max	Min	Max	Min	Max	Min	Max	Min	Max	Min/Max	Min	Max	Min	Max
Octadecene	1751		t	t	t		t		t				t						t					
Ethyl tetradecanoate	1774						t						t											t
Hexadecanal	1776	t	0.2		0.1	t	0.3		t		0.1		t	t	0.2		t			t	0.1	0.1		
Benzyl salicylate *	1790										0.3													
n-Octadecane	1800	0.1	0.3		0.1	0.2	0.3		0.3		0.1		0.4		0.2	0.1	0.6	0.1	0.2	0.1	0.1	0.2	t	0.3
Isopropyl tetradecanoate *	1803								t				t		t									
UI 23	1807	0.1	0.2				0.4		0.1			0.1	0.5	0.1	0.4		0.1		0.2	0.5	0.3	0.4		t
UI 24	1818		0.8	0.2	0.6	0.1	0.9		0.4		0.4		0.4	0.2	0.7	0.3	0.4	0.1	0.4	0.3	0.2	0.4		0.5
1-Hexadecanol	1821		0.9		0.2		t		t															
UI 25	1821		1.0	0.3	0.4	0.2	0.4		0.6		0.2	0.2	0.8	0.1	0.7	0.6	0.7	0.3	0.8	0.4	0.3	0.5		0.3
Nonadecene *	1825	0.6	2.1	1.0	1.6	0.8	1.9	0.3	1.4	0.4	1.1	0.1	1.8	0.5	1.9	1.0	1.5	0.9	1.7	1.2	0.7	1.2		1.3
Hexadecanol allyl ether	1827				0.1																			
n-Nonadecane	1900	4.0	9.3	4.2	7.4	3.7	8.8	1.4	5.6	2.0	5.1	1.8	14.7	2.1	11.8	6.4	25.6	3.9	11.0	6.9	3.7	9.6	0.6	6.2
Methyl hexadecanoate (methyl palmitate)	190		0.3				0.4		0.3		0.1		1.8		0.3									

Components	RI	Honeys																						
		Carob tree		Chest-nut		Eucalyp-tus		Bell Heather		In-cense		Laven-der		Orange		Rape		Raspber-ry		Rose-mary	Sunflow-er		Strawber-ry tree	
		Ct1-Ct5		C1-C2		E1-E5		H1-H6		I1-I4		L1-L8		O1-O9		R1-R2		Rb1-Rb2		Ro1	Sf1-Sf3		St1-St4	
		Min	Max	Min	Max	Min	Max	Min	Max	Min	Max	Min	Max	Min	Max	Min	Max	Min	Max	Min/Max	Min	Max	Min	Max
	4																							
Hexadecanoic acid (palmitic acid)	1908	1.0	4.9	0.3	0.8	0.5	2.8		2.7	3.3	6.5		2.1	0.9	5.4	4.4	5.4	2.3	7.7	7.2	4.7	13.4	1.9	5.5
Ethyl hexadecanoate (ethyl palmitate)	1936			0.1	0.2	t	0.6		0.5	0.3	0.5		1.6		0.4	0.4	0.9	0.5	0.8	0.8	t	1.1		0.3
1-Heptadecanol	1975		0.2		t		0.3						0.1						t		t	t		
n-Eicosane	2000		0.6	0.2	0.6	0.2	1.2		0.6	t	0.3		1.1	0.1	0.9	0.1	0.7				0.2	0.5		0.3
Octadecanal	2008		1.3				0.3						0.5					0.1	0.8					
UI 26	2016																				3.0	4.1		
trans-9-Octadecen-1-ol	2037								0.1															
Oleyl alcohol * [(*Z*)-octadec-9-en-1-ol]	2044				0.1		0.2									0.1	0.3					11.4		
Methyl linolenate	2064															2.1	3.0							
UI 27	2067		0.3				2.0							0.1	2.2		0.4		1.8	0.3	2.2	2.6		
Methyl linolelaidate	2095		1.1										3.1											
1-Octadecanol (stearyl alcohol)	2095		3.1		4.0		3.4		4.5		1.1		3.5				0.4							
Heneicosene *	2096	t	3.7		1.7		3.1		2.0		0.8		5.4	1.3	4.3	t	0.2		4.3	1.7	t	0.4	0.3	2.4

Components	RI	Honeys																						
		**Carob tree**		**Chest-nut**		**Eucalyp-tus**		**Bell Heather**		**In-cense**		**Laven-der**		**Orange**		**Rape**		**Raspber-ry**		**Rose-mary**	**Sunflow-er**		**Strawber-ry tree**	
		**Ct1-Ct5**		**C1-C2**		**E1-E5**		**H1-H6**		**I1-I4**		**L1-L8**		**O1-O9**		**R1-R2**		**Rb1-Rb2**		**Ro1**	**Sf1-Sf3**		**St1-St4**	
		**Min**	**Max**	**Min**	**Max**	**Min**	**Max**	**Min**	**Max**	**Min**	**Max**	**Min**	**Max**	**Min**	**Max**	**Min**	**Max**	**Min**	**Max**	**Min/Max**	**Min**	**Max**	**Min**	**Max**
Methyl oleate (methyl cis-9-octadecenoate)	2096		0.1				t		t				6.3											1.0
Methyl stearate	2096												1.0											
n-Heneicosane	2100	4.9	8.6	4.7	7.1	4.1	7.3	1.3	6.3	2.7	4.0	2.4	11.4	3.5	25.5	5.7	15.1	4.0	8.4	7.2	3.9	9.8	2.9	7.0
Methyl linoleate	2101						0.5						4.4											
Oleic acid (*cis*-9-octadecenoic acid)	2119	0.7	13.7	0.7	6.2	0.4	10.9	0.4	4.0	17.3	38.8	0.4	3.5	t	31.7		4.9	0.1	23.9		2.0	2.6	0.8	9.1
Ethyl linoleate (linoleic acid ethyl ester)	2137								4.4								2.1		2.1					
Linoleic acid (*cis*-9, *cis*-12-octadecadienoic acid)	2140		2.3				1.2		1.3		9.4		0.5						0.1	1.2				
Ethyl oleate (oleic acid ethyl ester)	2151		6.9	t	1.6		2.4		5.7		9.3		3.3		10.8	0.6	8.1	1.0	3.5	4.4		2.0		2.2
Acetic acid octadecyl ester (stearyl acetate)	2166												0.4											
Docosene	2170		2.1	t	0.8		1.0		0.3				2.3						0.4					
n-Docosane	2200	t	1.8	0.8	1.0	t	0.8		0.6		1.1		2.2	0.2	1.1	0.5	0.6	0.6	1.5		1.6	2.0		
n-Eicosanal	2200		0.6										2.2											
Labd-7-en-15-ol *	2202																		0.4					
Tricosene 1	226		3.9	3.4	3.9		4.0	1.4	3.8	2.1	2.4	2.4	4.1	0.8	4.1	1.5	2.8	3.2	4.0	3.6	0.1	2.0	0.2	4.5

Components	RI	Honeys																						
		Carob tree		Chest-nut		Eucalyp-tus		Bell Heather		In-cense		Laven-der		Orange		Rape		Raspber-ry		Rose-mary	Sunflow-er		Strawber-ry tree	
		Ct1-Ct5		C1-C2		E1-E5		H1-H6		I1-I4		L1-L8		O1-O9		R1-R2		Rb1-Rb2		Ro1	Sf1-Sf3		St1-St4	
		Min	Max	Min	Max	Min	Max	Min	Max	Min	Max	Min	Max	Min	Max	Min	Max	Min	Max	Min/Max	Min	Max	Min	Max
	4																							
Tricosene 2	2264	2.2	3.9	3.4	3.9		4.0	1.4	3.8	1.3	2.3	2.4	4.1	1.7	4.4	1.53	2.8	2.1	3.3	3.6	0.1	2.0	0.2	2.4
(Z)-9-Tricosene	2287		3.7				6.1																	
n-Tricosane	2300	10.9	29.3	20.0	21.0	15.5	16.9	6.0	16.9	7.5	11.0	12.2	20.6	9.4	18.0	13.4	21.0	14.8	15.2	20.7	14.6	22.9	1.2	14.2
UI 28	2355																				1.1	3.4		
3-meC23	2363	t	0.6	0.2	0.2	t	0.2		0.3		0.1		0.2		0.5	t	0.2	0.1	0.6	0.5			t	0.4
n-Tetracosane	2400	0.2	0.7	0.5	0.6	0.3	1.1		1.1	0.1	0.8		2.1	0.5	2.5	0.4	0.9	0.2	0.8	1.0	t	0.5		t
Pentacosene 1	2461	0.9	1.9		2.1	1.3	2.3	0.7	1.8	1.1	1.5	1.2	3.6	1.1	2.3	0.5	1.8	1.0	1.3	2.4	1.1	1.4	0.2	2.0
Pentacosene 2	2461	0.9	1.9	2.1	3.4	1.3	2.3		1.8	1.1	1.5		3.8	1.1	2.3	0.5	1.8	1.0	1.3	2.4	1.1	1.4	0.2	2.0
1-Docosanol	2498								1.6				1.3											
n-Pentacosane	2500	5.2	15.6	9.1	9.6	5.7	8.2	2.7	8.3	4.4	7.8	6.0	12.1	0.3	13.4	7.0	16.1	6.5	7.5	9.1	6.7	13.7	1.6	7.7
3-meC25	2560		0.3		t	0.2	0.5		0.8		0.6		0.7	0.1	0.8				0.9		0.2	0.5		
n-Hexacosane	2600	0.1	1.4	0.5	2.0	0.4	0.8		3.4	0.3	0.6	0.3	1.1	t	0.9	0.2	0.6	0.1	0.2	0.4	0.2	1.1	0.1	0.3
Heptacosene	2667	0.2	1.7	1.7	2.4	0.8	1.6	0.5	2.0	0.1	5.4	0.4	2.4		6.2	0.2	1.3	0.9	1.3	1.4	0.5	1.1		

Components	RI	Honeys																						
		Carob tree		Chestnut		Eucalyptus		Bell Heather		Incense		Lavender		Orange		Rape		Raspberry		Rosemary	Sunflower		Strawberry tree	
		Ct1-Ct5		C1-C2		E1-E5		H1-H6		I1-I4		L1-L8		O1-O9		R1-R2		Rb1-Rb2		Ro1	Sf1-Sf3		St1-St4	
		Min	Max	Min	Max	Min	Max	Min	Max	Min	Max	Min	Max	Min	Max	Min	Max	Min	Max	Min/Max	Min	Max	Min	Max
n-Heptacosane	2700	2.3	8.2		2.9	1.9	3.3	1.1	4.0	t	7.1	1.6	6.3	0.7	5.9	2.7	5.7	3.2	3.2	3.3	2.0	4.7	1.0	4.5
3-meC27	2743						0.2				1.0								0.3					
5-meC27	2746						0.3						0.2											
n-Octacosane	2800		0.7		4.6		0.1		0.4		0.3													
n-Nonacosane	2900		2.0				0.6		0.5	1.7	4.0		13	0.3	2.5	0.4	0.4		0.5	0.8	0.2	0.8	0.6	1.2
n-Hentriacontane	3100										0.3												0.1	1.1
**% Identification**		89.5	92.8	78.3	90.2	80.9	90.7	72.0	90.7	81.5	90.4	68.8	90.1	70.8	90.3	95.6	96.4	88.4	91.1	91.2	69.6	87.0	81.7	88.7
**Grouped components**																								
**Terpenes and derivatives**																								
Hemiterpene hydrocarbons						t	t	t	t			t	t					t	t					
Monoterpene hydrocarbons			0.4			t	0.4	t	1.2		0.3		0.4		0.5			t	0.2		t	0.2		0.1
Oxygen-containing monoterpenes		0.4	25.1	0.2	1.7	1.0	12.2	5.8	27.4	1.7	5.4		1.0	0.4	4.7	0.2	0.4	1.0	1.1	0.6	t	0.3	0.1	0.6
Sesquiterpene hydrocarbons			t		t		0.5		0.3		0.2		1.8		t		t		t		0.1	0.5		t
Oxygen-containing sesquiterpenes			t	0.6	1.0	0.5	1.8		0.5	t	0.5		0.8	t	1.0	t	t	0.8	1.3	t	t	t		4.2
Oxygen-containing diterpenes			t				t								0.1				0.4					
Apocarotenoids			0.6		0.1	t	2.3	0.1	3.7				t		0.1		t				t	0.1	5.2	44.3
**Amino acid derivatives and Phenylpropanoids**																								
Benzoic acids derivatives			0.3	0.4	1.2	t	1.1	0.4	2.6	t	0.7		1.1		0.2	t	t	t	0.2	0.1		t		0.3

Components	RI	Honeys																						
		Carob tree		Chestnut		Eucalyptus		Bell Heather		Incense		Lavender		Orange		Rape		Raspberry		Rosemary	Sunflower		Strawberry tree	
		Ct1-Ct5		C1-C2		E1-E5		H1-H6		I1-I4		L1-L8		O1-O9		R1-R2		Rb1-Rb2		Ro1	Sf1-Sf3		St1-St4	
		Min	Max	Min	Max	Min	Max	Min	Max	Min	Max	Min	Max	Min	Max	Min	Max	Min	Max	Min/Max	Min	Max	Min	Max
Phenylpropenes			0.9	0.1	0.5	t	0.4	t	1.1	t	0.3		1.1	t	0.3				0.2	t			t	t
Aromatic amino acid derivatives		0.2	1.1	2.2	2.2	0.2	3.1	1.9	25.2	1.0	3.1	3.5	9.9	0.9	2.3	0.1	0.4	1.2	4.0	1.0	0.1	1.1	2.0	18.6
**Fatty acids and derivatives**																								
Green leaf volatiles (GLV)		t	t	t	t	t	0.1	t	0.1	t	t	t	t	t	t	t	t	t	t	t	t	t		
Fatty acids		3.2	18.6	1.6	8.2	5.5	12.6	3.2	11.4	20.6	45.3	1.0	4.6	2.2	37.0	5.4	9.4	3.1	31.7	8.5	8.5	18.0	2.8	15.0
Alkanes		35.9	74.2	49.6	55.8	40.8	53.2	15.2	47.8	24.5	42.7	27.7	64.9	34.9	65.7	61.3	77.0	39.1	52.9	54.2	43.2	68.3	11.2	45.4
Methyl-branched hydrocarbons		0.2	0.8	0.2	0.2	0.4	0.8		0.9		1.3		0.8	0.3	1.2	t	0.2	0.4	1.5	0.5	0.2	0.5	t	0.4
Other fatty acid derivatives		13.1	24.4	18.6	22.8	15.7	22.6	10.2	25.2	10.7	17.4	16.1	27.5	8.8	28.5	12.6	24.9	13.1	26.7	22.6	6.6	23.4	2.0	18.2
**Carbohydrate derivatives**			2.4		t	0.7	2.0	0.1	2.8	0.1	0.7		1.1	t	0.7	t	0.1	t	0.6	0.1	0.1	0.2	t	0.9
**Nitrogen containing compounds**																							t	0.1
**Sulfur containing compounds**																t	t							
Others			t	0.1	1.1	t	0.2	0.4	3.5				0.4		t	t	t	t	t		t	t	t	1.4

RI: In-lab calculated retention index relative to C6-C31 *n*-alkanes on the DB-1 column; Min: Minimum. Max: Maximum. t: traces (< 0.05 %). * Identification based on mass spectra only. UI: unidentified compounds. MS (EI, 70 eV) *m/z* (Intensity ≥ 10%): *UI 8*: 41 (21), 44 (24), 55 (11), 67 (18), 68 (10), 77 (14), 79 (82), 81 (10), 91 (13), 93 (15), 94 (100), 95 (10). Ct: Carob tree. C: Chestnut. E: Eucalyptus. H: Bell heather. I: Incense. O: Orange. R: Rape. Rb: Raspberry. Ro: Rosemary. L: Lavender. S: Sunflower. St: Strawberry tree.
